# Correction of Severe Hallux Valgus Deformity Using a Percutaneous Metatarsal Distal Osteotomy

**DOI:** 10.1177/24730114251363448

**Published:** 2025-08-22

**Authors:** Jonas Müller, Gilles Dietrich, Igor Gossuin, Marc Prod’homme, Salah Dine Qanadli, Elyazid Mouhsine

**Affiliations:** 1 University of Lausanne UNIL, Faculty of Biology and Medicine FBM, Lausanne, Switzerland; 2 University Hospital of Vaud CHUV, Orthopedic surgery, Lausanne, Switzerland; 3 Lausanne West Medical Center, Lausanne, Switzerland; 4 Swiss Leman Institute of Traumatology and Orthopedics ITOLS, Lausanne, Switzerland

**Keywords:** hallux valgus, severe form, percutaneous, distal metatarsal osteotomy, transverse osteotomy, satisfaction, complications, associations

## Abstract

**Background:**

Severe hallux valgus (HV) presents a significant surgical challenge. Traditional methods are being questioned because of their invasiveness, whereas recent minimally invasive techniques raise concerns because of their associated complications. This study evaluates the percutaneous distal metatarsal osteotomy technique, generally found to be effective for mild-to-moderate cases, and tests the hypothesis that it provides effective clinical and radiologic correction for severe deformities.

**Methods:**

This retrospective study analyzed 116 feet that underwent percutaneous distal metatarsal transverse osteotomy with lateral soft tissue release and provisional Kirschner wire fixation, with a mean follow-up of 27.1 months, limited to severe cases (hallux valgus angle [HVA] > 40 degrees). Radiologic assessments included preoperative and postoperative measurements of HVA, intermetatarsal angle (IMA), distal metatarsal articular angle, sesamoid position, first metatarsophalangeal (MTPI) joint congruency, metatarsal length, and sagittal position. Clinical evaluations used the AOFAS scale, documenting the recurrence rate, the nature of complications, reoperations, and the association between them. Patient satisfaction was assessed through self-reported evaluations.

**Results:**

Significant improvements were noted for HVA (median correction from 43.1 to 14.6 degrees) and IMA (median correction from 17.2 to 8.5 degrees). The metatarsal was shortened by 5.4 mm. There was a notable reduction in the degree of sesamoid displacement and MTPI congruency. Sagittal position remained unchanged in 85.3%. The median AOFAS score improved from 44.0 to 90.5, well above the clinically significant improvement threshold, and 87.9% of patients were satisfied or very satisfied. We recorded no major complications and minor complications at a rate of 35.3%. Reoperation rate was 14.7%, primarily due to exostoses. Significant associations were found between postoperative sesamoid position and clinical outcome, and between reoperation rate, exostosis, and MTPI congruency, emphasizing the importance of correcting these parameters. Recurrence rate was 6%. Patient satisfaction was associated with reoperation and complications, but not with radiologic parameters.

**Conclusion:**

Percutaneous distal metatarsal osteotomy achieved substantial correction of severe hallux valgus with significant improvements in angular measurements, high patient satisfaction (87.9%), and no major complications. Although the technique shows promise as a less invasive alternative with comparable radiographic outcomes, the 14.7% reoperation rate (primarily for exostoses) and 6% recurrence rate must be considered. Prospective comparative studies are needed to establish its role relative to other surgical approaches for severe deformities.

**Level of Evidence:** Level IV, retrospective case series.

## Graphical abstract

**Graphical Abstract:**
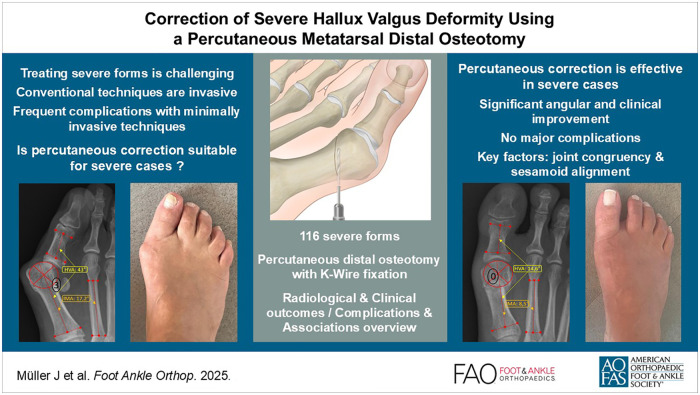

## Introduction

Severe hallux valgus (HV) deformity poses a significant clinical challenge because of the lack of consensus on the most appropriate surgical management strategy.^41,46,49^ Traditional approaches, such as open proximal osteotomies, optionally combined with arthrodesis, have been widely used. However, these methods are frequently questioned because of their invasiveness and potential complications.^24,26,54,56^ Recently, minimally invasive surgery (MIS) approaches have been applied to severe forms. These third- and fourth-generation techniques have specific limitations mainly related to soft tissue (eg, scars, nerve irritation, tendon injury) and osteosynthesis material complications (eg, painful hardware).^19,35,62^ In contrast, percutaneous distal osteotomy, often referred to as “2nd generation MIS,” has proven effective for mild to moderate forms, offering equivalent radiologic and clinical outcomes, with specific advantages such as no scarring and no residual osteosynthesis material.^6,25,58^ Nevertheless, this approach is not commonly used for severe cases, being overshadowed by more invasive procedures. Additionally, severe cases often involve elderly patients with multiple comorbidities, necessitating the use of the least invasive approach possible. Given that severe forms differ from milder ones only in terms of severity, with identical pathophysiological mechanisms,^20^ we hypothesize that this less invasive technique—whose efficacy has been demonstrated through decades of clinical experience and research—may be appropriate and effective for managing severe cases. This paradigm to treat severe forms remains largely underexplored in current literature, with existing studies analyzing only a limited number of parameters and patients, and failing to clearly define the reported complications. Given this context, the primary objective of this publication is to fill the current gap in the literature by proposing a rigorous study that precisely details the clinical and radiologic outcomes of the percutaneous distal osteotomy technique with Kirschner (K)-wire stabilization, specifically adapted for severe cases. Secondarily, this study also provides a comprehensive analysis of complications, patient satisfaction, and the correlation between all measured parameters. This would provide practitioners with a better understanding of the variables that influence outcomes, thereby optimizing the management of this complex pathology.

## Material and Methods

This retrospective study comprised 116 feet from 95 patients who underwent surgery at a single center between August 2018 and October 2021. Data were collected up to 3 months preoperatively and at the final follow-up. Inclusion criteria were as follows: severe hallux valgus angle (HVA >40 degrees),^14^ acquired, unilateral or bilateral involvement, failure of conservative treatment, alongside a signed consent form for medical study use. Exclusion criteria comprised congenital origins, advanced degenerative disorders of the first ray (such as osteoarthritis, rheumatic diseases), other specific hallux pathologies (rigidus, varus), recurrences, subsequent corrections, incomplete follow-up (minimum follow-up: first postoperative consultation), and/or patient’s desire to withdraw from the study. The procedures were performed by a single, highly experienced surgeon. The principal author, not belonging to the surgical team and assisted by the coauthors, conducted the collection and interpretation of clinical and radiologic data, whereas statistical analysis was performed by an independent organization.

The clinical evaluation was based on the pre- and postoperative American Orthopaedic Foot & Ankle Society (AOFAS) metatarsophalangeal-interphalangeal (MTP-IP) scale.^28^ We documented the rate and nature of intraoperative and postoperative complications, classified as major and minor, along with reoperations, which were recorded up to the point of surgical revision. An initial HVA corrected to less than 20 degrees that exceeded 20 degrees at the final follow-up was considered a recurrence.^29,44^ Patient satisfaction assessments, which reflect overall satisfaction with respect to the surgical correction, were based on patient self-reporting and conducted at the end of treatment using a 4-degree scale: very satisfied, satisfied, unsatisfied, very unsatisfied.

Radiologic assessments were performed pre- and postoperatively using anteroposterior (AP) and lateral weightbearing radiographs, measured with the Synedra radiology software, according to AOFAS recommendations,^15,50^ including HVA, intermetatarsal angle (IMA) and distal metatarsal articular angle (DMAA). Additional metrics recorded in the AP plan included the congruence of the first metatarsophalangeal joint (MTPI) (Figure 1), spanning 3 stages (congruent, subluxated, luxated),^13,14^ positions of the sesamoid bones using a 4-position scale (based on the tibial sesamoid, with grades 0 and 1 considered normal) (Figure 2),^50^ first metatarsal (MT1, M1, or MI) length,^40^ and sagittal metatarsal (MT) head positioning (neutral, dorsal, plantar) (Figure 3).

**Figure 1.**
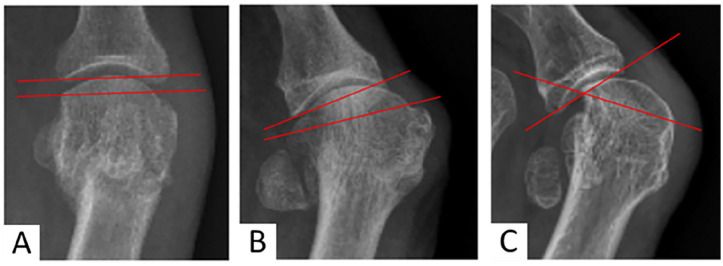
Anteroposterior weightbearing view illustrating the 3 stages of metatarsophalangeal joint alignment, defined by the relationship between the articular surfaces relative to the reference articular margin (red lines). (A) Congruent; lines are parallel. (B) Subluxated; lines are convergent and intersect outside the joint line. (C) Luxated; lines are convergent at the level of the joint line.

**Figure 2.**
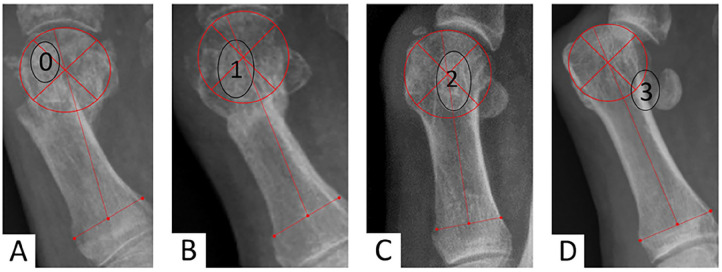
Anteroposterior weightbearing view illustrating the 4 grades of medial sesamoid luxation relative to the metatarsal axis. The axis is defined by a line connecting the midpoint of the proximal diaphysis with the center of the head, allowing for measurement of displacement even after a subcapital osteotomy. (A) Grade 0. (B) Grade 1. (C) Grade 2. (D) Grade 3.

**Figure 3.**
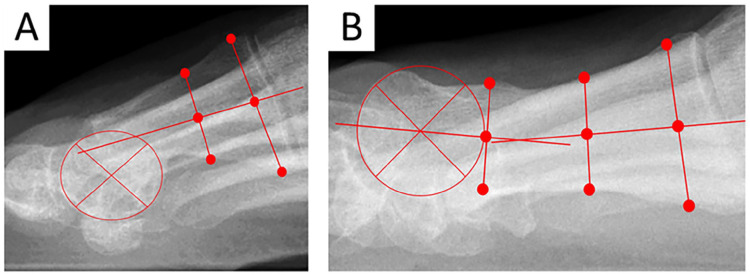
Lateral weightbearing radiograph illustrating sagittal postoperative displacement of the metatarsal head, measured by a significant loss of alignment between the center of the head and the metatarsal axis. (A) Plantar displacement. (B) Dorsal displacement.

Overall, the surgical technique aligns with a second-generation MIS approach, in which the osteotomy is derived from the Bosch technique. All corrective procedures were executed through percutaneous portals.^51^ Regardless of the sesamoid position or MTPI congruence, a lateral soft tissue release by sectioning the phalangeal insertion of the adductor hallucis tendon (using a Beaver blade) was systematically performed (Figure 4A). Through a second medial percutaneous portal, an “exostosectomy” of the medial eminence was conducted using a thin 2-mm burr by performing “windshield wiper” movements without violating the capsule. This was followed by drainage of the bone paste through digital pressure and thorough rinsing via the portal. A transverse slightly oblique, 2-plane subcapital MTI osteotomy was then executed from plantar to dorsal (Figure 4B). Depending on the need to correct the length of the first ray, the osteotomy was inclined in the transverse plane. In the sagittal plane, the osteotomy was also slightly oblique (oriented from plantar and proximal to dorsal and distal) to prevent the ascent of the MT head. A K-wire (2 mm) was inserted anterogradely and subcutaneously, lateral to the hallux to a distal skin perforation. Manual repositioning of the MT head involved a combination of distal derotation (or supination) and lateral translation. Subsequently, the correction was stabilized by retrograde insertion of a K-wire into the metatarsal shaft and impacted at its base (Figure 4C). Further corrections could be made depending on the K-wire’s placement in the sagittal plane; positioning above the sagittal axis of the first metatarsal lowers the head, while positioning below induces a slight ascent. The coronal orientation of the wire in the shaft depends on the degree of lateral translation of the head required to correct the deformity. The wire can be positioned parallel to the axis, convergent, or divergent, allowing to correct the tilt of the metatarsal articular surface (Figure 4D). Additional procedures such as Akin osteotomy or shortening of other rays were performed as needed. The positions of the K-wire, sesamoids, MTPI congruency, and sagittal position of M1 were systematically verified under fluoroscopy at the procedure’s end. A slightly corrective dressing was routinely applied, and each use of the burr was conducted under constant irrigation to prevent thermal skin necrosis. The standardized postoperative protocol included immediate ambulation in full weightbearing with a rigid flat sole for 4 weeks, with follow-up dressing changes weekly until wound healing. The K-wire was removed at the fourth postoperative week, followed by a gradual resumption of daily and recreational activities.

**Figure 4.**
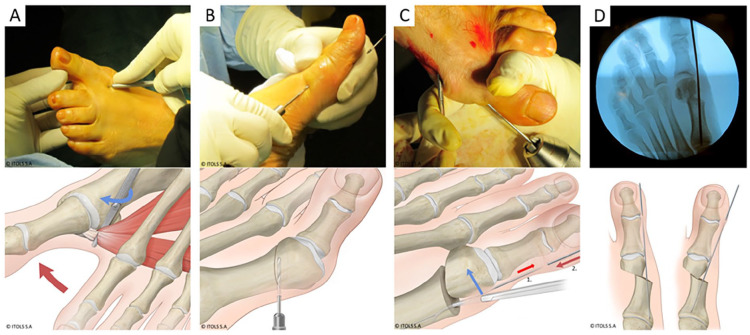
Pictures (above) and corresponding illustrations (below) of the key steps of the surgical technique. (A) Percutaneous tenotomy of the adductor hallucis tendon. Perioperative picture shows forced abduction of the hallux to tension the tendon and facilitate its sectioning. The sudden loss of resistance indicates a complete tenotomy. Illustration shows the phalangeal detachment of the tendon using a Beaver blade without opening the joint capsule. The red arrow shows the forced abduction movement of the hallux. The blue arrow indicates the movement of the Beaver for targeted tenotomy. (B) Metatarsal osteotomy. Intraoperative image shows the subcapital entry point of the burr, starting from the plantar side. Illustration shows the slight obliquity of the transverse osteotomy in 3 planes, which allows limiting the ascent of the head, lengthening the metatarsal if needed, correcting the rotation and laterally translating the head. (C) Correction of the deformity and stabilization of the osteotomy by inserting the K-wire into the metatarsal shaft. Intraoperative picture shows this combined maneuver and the placement of the forceps through the osteotomy portal. Illustration shows the mosquito-type forceps in the metatarsal shaft at the osteotomy level. The wire is initially introduced through the osteotomy portal and pushed at the metatarsal head level in an anterograde direction (red arrow no. 1) into the soft tissues lateral to the metatarsal head and phalanges until it exits distally. The wire is then mounted on a T-handle with a chuck, allowing it to be pushed retrogradely into the metatarsal shaft (red arrow no. 2). At the same time, the Mosquito clamp provides support on the metatarsal head to translate it laterally (blue arrow) while guiding the pin into the shaft. Note also the reduction of the metatarsophalangeal joint by this maneuver. (D) Perioperative final fluoroscopy to control the degree of correction as well as the positioning of the wire. Illustrations showing different options for coronal orientation of the wire in the shaft, depending on the degree of lateral translation of the head required to correct the deformity, but also to correct the tilt of the metatarsal articular surface. The wire can be positioned parallel to the axis, divergent (left image) or convergent (right image).

All statistical analyses were performed using IBM SPSS 29.0.1.0 software. The alpha risk was set to 5%, and 2-tailed tests were employed. Differences with a *P* value less than .05 were considered significant.

## Results

The demographic and clinical characteristics of the study population are summarized in Table 1, providing a foundational overview.

**Table 1.** Demographic and Clinical Characteristics of the Study Sample.

Characteristic	Value	Notes
Total feet / patients, n	116/95	21 cases bilateral
Sex distribution, female/male, %	89.7/10.3	—
Foot laterality: right/left, %	50/50	—
Mean age at surgery, y	67.8	SD: ±9.7
Mean follow-up, mo	27.1	SD: ±11.2
Comorbidities, % (n)	28.4 (27)	Mainly type 2 diabetes
Primary surgical indications, % (n)	80.2 (93)	Pain at MTP and medial eminence
Secondary indications, % (n)	19.8 (23)	Walking, footwear, physical activity limitations
Associated deformities, % (n)	61.2 (61)	58.6% claw and hammer toes
Additional corrective interventions, % (n)	75.9 (88)	39.6% Akin P1, 16.3% claw and hammer toes correction

Abbreviations: MTP, metatarsophalangeal joint; P1, first phalanx.

Building on the patient characteristics, the radiographic findings at final follow-up (Table 2 and Figure 5) revealed significant improvements. The median pre- and postoperative HVA (degrees) were 43.1 (SD 4.95) and 14.6 (SD 8.2), respectively, with a median correction of −29.0 (SD 8.6) (paired *t* test, *P* = .001). The median pre- and postoperative IMA (degrees) were 17.2 (SD 3.9) and 8.5 (SD 4.0), respectively, with a median significant correction of −8.3 (SD 3.8) (paired *t* test, *P* = .001). Whereas no significant change was observed for the DMAA, with median pre- and postoperative (degrees) of 10.8 (SD 8.8) and 11.0 (SD 7.9), respectively, and a median change of 0.7 (SD 0.7) (paired *t* test, *P* = .21). The length of M1 was shortened by a median −5.4 mm. Correction of high-grade sesamoid displacement was statistically significant, particularly in grade 3 cases (paired Wilcoxon test, *P* > .001). Additionally, MTPI congruency significantly improved, with a notable decrease in luxated forms (paired Wilcoxon test, *P* > .001). The sagittal alignment remained stable in 85.3% of cases, with 9 feet showing a dorsal residual position and 8 feet a plantar residual position.

**Table 2.** Radiographic and Clinical Outcomes.^a^

Outcome	Preoperative	Postoperative	Difference	*P* Value
HVA, degrees	43.1 (4.9)	14.6 (8.2)	−29.0 (8.6)	<.001
IMA, degrees	17.2 (3.9)	8.5 (4.0)	−8.3 (3.8)	<.001
DMAA, degrees	10.8 (8.8)	11.0 (7.9)	0.7 (0.7)	.21
M1 length, mm, mean (SD)	60.75 (4.5)	55.60 (4.3)	−5.4 (3.1)	<.001
Sesamoid high grade (2 and 3), %	98.3	43.1	↓ 55.2	<.001
Sesamoid grade 3 only, %	81.9 ± 3.6	6.0 ± 2.2	↓ 75.9	<.001
MTPI congruency (luxated), %	95.7 ± 1.9	5.2 ± 2.1	↓ 90.5	<.001
Sagittal M1 head position (neutral), %	100	85.3	↓ 14.7, 7.7 dorsal, 6.8 plantar	.09
AOFAS total score (points), median (IQR)	44.0 (20.0)	90.5 (10.0)	↑ 47.0 (17.2)	<.001

Abbreviations: AOFAS, American Orthopaedic Foot & Ankle Society metatarsophalangeal-interphalangeal scale; HVA, hallux valgus angle; IMA, intermetatarsal angle; IQR, interquartile range; DMAA, distal metatarsal articular angle; M1, first metatarsal; MTPI, first metatarsophalangeal joint.

a Unless otherwise noted, values are mean (SD).

**Figure 5.**
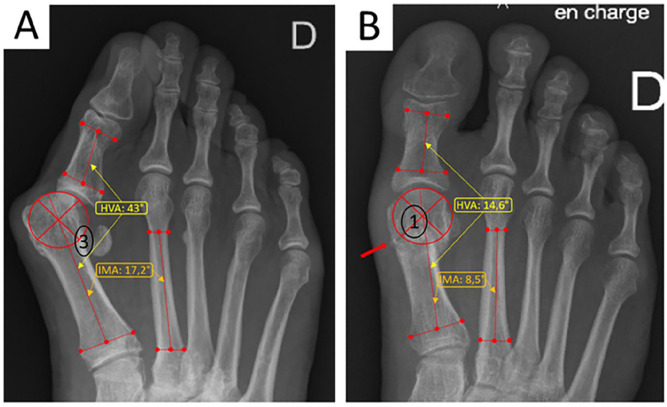
Radiographic illustration of pre- and postoperative median deformities in our series. (A) Weightbearing preoperative radiographs showing the median angular deformations of HVA and IMA, the mean sesamoids grade (grade 3), and the luxation position of the MTPI. (B) Weightbearing postoperative radiograph showing the consolidated subcapital osteotomy (red arrow), the median correction of the HVA and IMA, the congruence of the MTPI, and the reduction of the sesamoids (grade I).

These radiographic improvements were mirrored by encouraging clinical outcome. The median pre- and postoperative AOFAS total scores (X/100) were 44.0 (IQR = 20.0) and 90.5 (IQR = 10.0), respectively, with a median significant improvement of 47.0 points (IQR = 17.2) (paired *t* test, *P* = .001), covering all subitems (Table 2). At the end of the follow-up, the majority of patients (87.9%) were satisfied or very satisfied (55.2% and 32.8%, respectively), whereas only 10 patients were unsatisfied and 1 was very unsatisfied.

Alongside the clinical benefits, no major postoperative complications were observed (Table 3). The rate of minor complications was 35.3%, predominantly for exostoses (Figure 6), and 11 cases of undercorrection. The reoperation rate was 14.7%, primarily for exostoses. Although recurrence occurred in 7 cases (6%), all were asymptomatic and did not require additional treatment.

**Table 3.** Summary of Complications and Reoperations.

Complication	Number of Feet	Percentage	Notes
Major	0	0	None observed
Exostosis	24	20.7	Residual ossified bone in medial soft tissue
Undercorrection	11	9.4	Lack of clinical/radiologic improvement
Stiffness	3	2.6	<30 degree range of motion
Pronation of hallux	2	1.7	Asymptomatic
Intraoperative (misplaced osteotomy)	1	0.8	Placement corrected
Overcorrection (hallux varus)	1	0.8	Asymptomatic
Reoperation			
For exostosis	11	9.4	Mini-invasive excision
For undercorrection	3	2.6	New initial procedure
For stiffness	2	1.7	Mobilization under narcosis

**Figure 6.**
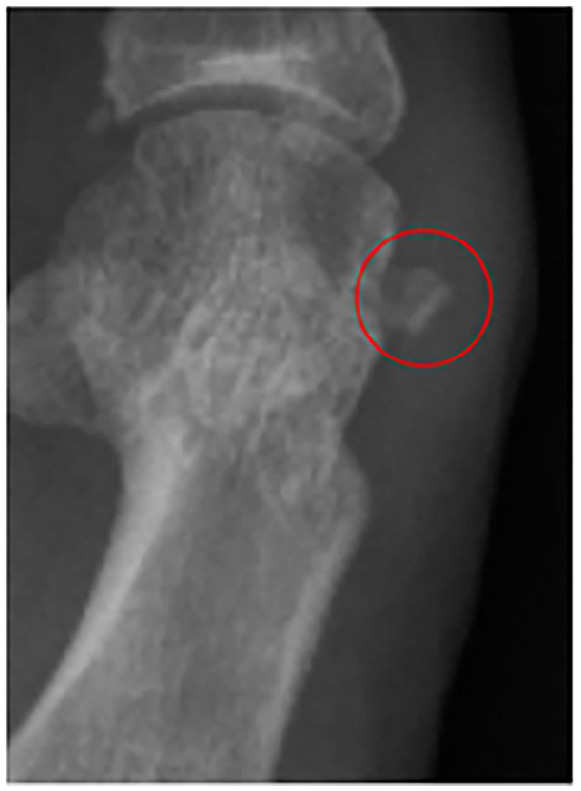
Postoperative radiograph illustrating an exostosis (into the red circle).

To better understand the interplay between outcomes and variables, we next explored associations (Table 4). There were no significant differences between groups in terms of baseline demographics or comorbidities. However, a significant relationship emerged between postoperative HVA and MTPI congruency (Dunn-Bonferroni test, *P* = .011), with pairwise analysis highlighting differences between subluxated and congruent joints. Hence, higher postoperative HVA was associated with an increased likelihood of MTPI subluxations. Similarly, the sagittal position of the M1 head was significantly associated with postoperative HVA (Fisher exact test, *P* = .034), particularly when comparing plantar and dorsal orientations. Sagittal plane malalignments were therefore associated with higher postoperative HVA values. Interestingly, postoperative HVA was not correlated with any preoperative radiologic parameters, nor with the incidence of complications or reoperations.

**Table 4.** Summary of the Associations Between Several Parameters of interest.

Variable	Associated Parameter	Finding	*P* Value
Postop HVA	MTPI congruency (congruent/subluxated/luxated)	Significant association Higher HVA if MTPI incongruence	.011 (subluxation vs congruence: 0.002)
Postop HVA	Sagittal M1 head position (neutral/plantar/dorsal)	Significant association with Plantar	.034 (pairwise: plantar vs dorsal, .026)
Postop HVA	Preop radiographic parameters, complications, reoperations	No association	—
Undercorrection	Postop high-grade sesamoid position (grades 2 and 3)	Significant association	<.001
Undercorrection	Other pre- and postoperative radiographic parameters	No association	—
Reoperation	Exostoses, Postop MTPI subluxation/luxation	Significant association	<.001
Patient satisfaction	Complications and reoperations	Significant association	<.001
Patient satisfaction	Radiographic parameters and AOFAS postop scores	No association	—
Postop AOFAS	Complications and reoperations	Significant association	<.001
Postop AOFAS	Sesamoid grades 0-3	Significant association with higher grades	.008 (pairwise: grade 0 vs 1: .01 grade 0 vs 2: .001 grade 0 vs 3: .011)
Postop AOFAS	Other postop radiographic parameters	No association	—

Abbreviations: AOFAS, American Orthopaedic Foot & Ankle Society metatarsophalangeal-interphalangeal scale; HVA, hallux valgus angle; M1, first metatarsal; MTPI, first metatarsophalangeal joint.

Focusing specifically on the subgroup with undercorrection, an association was found solely with postoperative high grade of the sesamoids (Fisher exact test, *P* < .001). No relation was found with other preoperative and postoperative radiologic parameters. Specifically, no association was found without preoperative HVA greater than 50 degrees and postoperative HVA greater than 20 degrees.

Among the reoperated group, findings pointed to a significant association with both exostosis (Fisher exact test, *P* < .001) and the presence of postoperative subluxation or luxation (Fisher exact test, *P* = .021). No relation was found with other postoperative radiologic parameters.

When analyzing patient satisfaction, the rates of complications and reoperations were significantly lower in the group of patients who reported being satisfied to very satisfied (Fisher exact test, *P* < .001). No differences related to radiologic parameters and postoperative scores were observed across the groups.

Finally, regarding the postoperative AOFAS score, a clear and statistically significant inverse relationship emerged in relation to the presence of complications and reoperations. The median postoperative total AOFAS score was 82.0 (IQR 15.0) in patients with postoperative complications and 95.0 (IQR 5.0) in those without (median difference = –13.0; Mann-Whitney test, *P* < .001). Similarly, the median score was 75.0 (IQR 12.0) in reoperated patients compared with 93.0 (IQR 7.0) in non-reoperated ones (median difference = –18.0; Mann-Whitney test: *P* < .001). No postoperative radiologic parameters were associated with the AOFAS score, except for the position of the sesamoids in high-grade cases (Fisher exact test: *P* < .001). Median postoperative AOFAS scores were higher in low-grade cases.

## Discussion

Key findings from our study include significant improvements in the HVA and IMA, high patient satisfaction rates, and the identification of a specific complication associated with this technique. The study also emphasizes the importance of correcting sesamoid position and MTPI joint congruency to optimize clinical outcomes. Given the growing interest in minimally invasive techniques, we believe this manuscript will be of particular interest to surgeons proficient in percutaneous approaches. Our findings contribute to the advancement of surgical strategies for severe HV by offering an effective alternative that reduces surgical trauma and supports faster recovery. This work adds to the international literature by presenting a large series managed with a strictly percutaneous technique without permanent fixation, clearly outlining both its advantages and limitations. It addresses a notable gap in the field, as few studies have focused exclusively on the management of severe hallux valgus using this approach.

Distal osteotomy provides radiologic correction comparable to proximal and advanced MIS techniques, making it suitable for severe deformities, with MTPI congruence emerging as a key factor influencing reoperation rates. The significant correction of the HVA and IMA angles demonstrates that distal osteotomy has corrective power comparable to proximal osteotomies, as previously demonstrated for moderate forms.^46,52^ Thus it is suitable for advanced severe deformities despite high preoperative angles. Moreover, the radiologic corrective power is equivalent to that obtained with third- and fourth-generation MIS techniques applied to severe forms.^35,62^ The corrective efficacy can be explained by the fact that transverse (and slightly oblique) osteotomies are oriented in a way that allows for free displacement of the metatarsal head in multiple planes. Additionally, the K-wire provides an added corrective force. This derotation is also observed radiologically through the reduction of the MTPI joint. Notably, the correction of these angles primarily influences the congruence of the MTPI, which in our series was a factor affecting the reoperation rate, rather than satisfaction and function, as previously demonstrated.^21,34,43^ Regarding the DMAA, although it is often measured in many studies and appears to reflect the pronation of the MT, our results are inconclusive. The unreliability of this parameter is largely due to considerable interobserver variability, particularly in postoperative measurements, where accurately defining the boundaries of the articular surface proves challenging. This results in marked discrepancies in assessment and a lack of statistically significant reproducibility, an issue similarly noted in this article.^9^

The correction of MTPI joint congruence and sesamoid position are 2 major parameters that must be considered during surgical correction. Severe HV is a 3-dimensional deformity, predominantly characterized by MT pronation and lateral luxation of the sesamoids,^27,39^ as also observed by the predominantly high preoperative grades in our series. Furthermore, these 2 conditions are linked and affect the congruence of the MTPI joint.^11,26^ The association between insufficient correction of the sesamoids and the congruence of the MTPI, which is prevalent in cases of undercorrection, illustrates this point. Furthermore, the correlation we have found between the position of the sesamoids and functionality underscores the importance of correcting this parameter, aligning with these observations.^4,8,32,53,54^ This aspect also likely contributes to influence the recurrence rate,^26,45^ which will be the subject of a specific publication after a more extensive follow-up.

Severe forms very often present with retracted sesamoids and a dislocated MTPI joint; therefore, percutaneous correction should combine a lateral release with a “derotational” osteotomy. This is why a 3-dimensional correction technique, with an intraoperative focus on sesamoid reduction and restoration of MTPI joint congruence, is essential.^16,21^ The severity of sesamoid luxation in severe cases justifies the necessity of systematically performing a lateral release.^32,59,61^ In severe cases, the sesamoids are sometimes retracted and incompletely realigned, making a combination of percutaneous lateral release with an osteotomy necessary.^11,60^ Despite showing improvement in the position of the sesamoids, this correction remains partially incomplete, highlighting a limitation of the percutaneous approach, which does not allow for radical correction of sesamoid retraction. Consistent with several studies, correcting pronation through derotation (or supination) of the metatarsal head helps realign the MTPI.^16,18,36,55,56^ The results of our technique, which combines a “derotational” osteotomy and a lateral release, enable the correction of MTPI congruency.

Correcting first metatarsal rotation is crucial for achieving effective 3-dimensional realignment in HV and optimizing surgical outcomes. Correction of the MT1 head rotation is achieved through hallux mobilization following osteotomy (joystick effect) and by the placement of a fixation pin (see Methods: Description of Surgical Technique), which stabilizes the correction. An additional pin may be (rarely) inserted if intraoperative fluoroscopic assessment reveals persistent malalignment or instability. To evaluate correction of MT1 pronation, we primarily assess the restoration of MTP1 joint congruence and the disappearance of the “lateral round sign”—an AP radiographic marker indicative of metatarsal pronation. To a lesser extent, correction of the medial sesamoid is also considered, although it may remain retracted in cases of severe deformity.^18,27,44^ Three-dimensional correction of HV, particularly through the correction of M1 rotation (which influences other parameters such as sesamoid position and MTP joint congruence)^4^ is now clearly recognized in the literature as a key factor impacting recurrence, clinical outcomes, and patient satisfaction.^44,45,53-56^

A transverse subcapital osteotomy is the most suitable approach for achieving 3-dimensional correction in severe HV, despite a risk of malposition in the sagittal plane, which, however, has no clinical impact. The literature identifies 2 primary types of distal M1 osteotomies: those following the Chevron method and those derived from Bosch’s original technique, which involves a transverse subcapital cut. The technique used in this study is the latter. Its long-term efficacy has been demonstrated for moderate deformities,^5,23^ and it presents outcomes comparable to those for less severe deformities.^37,62^ Moreover, this osteotomy allows for correction in 3 dimensions through translation and derotation, making it more suitable for a “derotational” correction,^4,10,45,56^ as well as for improved correction of sesamoid position.^10,45^ Sagittal malunion is reported as a specific complication associated with transverse osteotomies,^10^ which was indeed observed in our study, was however unrelated to patient satisfaction or reoperation rates. Furthermore, the cases of sagittal malunion do not appear to be significant enough to result in a negative clinical outcome, because, interestingly, no transfer metatarsalgia was observed.

Provisional K-wire fixation for severe HV provides a corrective effect, ensures stable and cost-effective correction, contributes to favorable outcomes, and avoids the disadvantages associated with permanent implants. K-wire offers the key advantage of leaving no permanent implant. The painful hardware is often described as a major complication with a significant clinical impact and which leads to the need for reoperation,^19,35^ and this is inherent in the third- and fourth-generation MIS technique, which does not exist in a technique employing provisional osteosynthesis. The absence of implants also limits the cost of this surgery and simplifies the technical facilities. In addition, the pin exerts a corrective force that a screw cannot. However, it is important to implant a sufficient pin diameter (2 mm minimum) to make the stabilization of the osteotomy sufficiently rigid. No fractures at the osteotomy site or pin migration were observed. The sufficient biomechanical stability of transverse osteotomies could explain this observation.^2^ In our technique, the stability is reinforced by applying a slightly oblique osteotomy in the sagittal plane, contributing to make definitive osteosynthesis unnecessary. These factors explain our much more favorable results for this percutaneous surgery technique with K-wire fixation than a study published in 2007.

The significant shortening observed post-osteotomy had no clinical impact and was consistent with rates reported in the literature.^42^ No influence on clinical outcomes and patient satisfaction has been found, nor any complications such as transfer metatarsalgia. The absence of exceeding the proposed shortening threshold of >5.8 mm,^1,43^ and the occasional shortening associated with adjacent rays, are likely favorable factors.

Postoperative AOFAS scores showed significant improvement, and patient satisfaction was high, though both measures rely on nonvalidated scales. The improvement observed in the postoperative AOFAS clinical score far exceeds the minimal clinically important difference threshold of 29 points.^7,30,57^ However, this nonvalidated (but commonly used in the literature) score primarily represents the clinician’s perspective. The lack of relation between the clinical score and postoperative radiologic parameters, except for the position of the sesamoids, reinforces this previous observation^40^ and highlights the importance of surgically correcting this parameter. Patient-reported outcome of satisfaction is the best way to assess treatment success from the patient’s perspective,^47^ and shows here a comparable rate to those in literature.^3^ Nevertheless, the satisfaction scale used in this study has not been validated; therefore, the findings should be interpreted with this limitation in mind and considered primarily as indicative.

Our study reported no major complications, supporting the safety of the percutaneous approach when performed by experienced surgeons. Complications associated with percutaneous approaches compared to open ones are often subject to controversy. Furthermore, the arbitrary definition of complications, when reported, makes comparing the nature and rate between studies extremely difficult. Our minor complications are better defined as suboptimal radiographic results. The absence in our study of major complications such as infections, nonunion, osteonecrosis, or neurologic and tendinous lesions aligns with numerous studies that have demonstrated safety and equivalence.^12,22,23,25,31,33,38^ Continuous irrigation during the use of the burr prevents necrosis of soft tissues,^62^ as was not encountered in this study. The absence of infection in our series may be explained by continuous irrigation of the portals during the surgery, and strict postoperative follow-up with regular dressing changes. Moreover, we did not observe any complications due to comorbidities, such as diabetes and vascular disease, attributed to a no-incision approach that minimizes trauma to soft tissues.^23^ It is clear that percutaneous surgery requires a long learning curve, and the surgeon’s experience significantly influences the outcomes.^24^

Undercorrection had minimal clinical impact, with rare revisions yielding satisfactory outcomes and no associated complications. We arbitrarily classified undercorrection as a minor complication, as the clinical repercussion is minimal, with only 2.5% requiring revision surgery, the reason for which was a lack of improvement in preoperative symptoms. The revision surgery consisted of an identical procedure to the first, with particular emphasis on reducing the MTPI. The reoperations were not associated with any complications and resulted in satisfactory clinical and radiologic correction.

A specific complication of this percutaneous technique is medial soft tissue exostosis, which increases reoperation rates but is minor and easily preventable. We highlight a complication not previously described, which is the presence of exostosis in the medial soft tissues, arising from residual bone paste. Most exostoses are located at the level of the exostosectomy, and more rarely at the osteotomy site, as they are more easily removed through the percutaneous portal during rinsing. This is a complication specific to this percutaneous technique and had a major impact on the rate of reoperation. Although revisions are a benign intervention focused on soft tissues, consisting of an excision via a mini-incision with a simple wound monitoring postoperative protocol, they have an indirect impact on patient satisfaction. However, recovery from this exostosis removal was always uncomplicated and, therefore, classified as a minor complication. The meticulous expulsion of the bone paste and irrigation of the portal can easily mitigate this specific complication.

The recurrence rate is acceptable but requires long-term follow-up for accurate interpretation. One of the most discussed aspects in the literature concerns long-term recurrence. Our 6% recurrence rate is slightly lower than those reported in studies focusing exclusively on severe cases (7% for Lewis et al,^35^ 6.6% for Neufeld et al,^41^ 15.6% for Seki et al^48^). However, it remains slightly higher than the 4.9% recurrence rate reported by Barg^3^ in his systematic review. The recurrence rate observed in our study should be interpreted in light of several limitations. First, it is based on short-term follow-up (<5 years), a major limitation also highlighted in this meta-analysis and systematic review,^29^ which assessed long-term recurrence and reported higher rates. Furthermore, severe deformities require correction of larger initial angles. Although current techniques offer substantial corrective power, the postoperative HVA values typically fall within the range associated with mild to moderate deformities, as illustrated by our findings. Therefore, recurrence rates should be interpreted in relation to the severity of the initial deformity. Additionally, a standardized definition of recurrence specific to severe HV is needed. Many studies apply different cut-off values, mixes of degrees of severity, which significantly influence reported recurrence rates,^29^ and often fail to clearly define the measurement criteria used.^19,29^ Although the initial anatomic alignment can be maintained in the medium term,^17,18^ longer studies are needed to confirm these observations. This aligns with our proposal to follow these patients over an extended period to better understand the recurrence rates associated with this method.

The technique meets essential goals of function, mobility, and surgical reversibility. Ultimately, beyond the correction of specific radiologic parameters, which have little impact on the patient’s experience, an optimal surgical technique must achieve the following objectives: maintaining the mobility of the MTPI joint, preserving the normal weightbearing pattern of the forefoot, and providing a reasonable backup solution if the surgery fails.^14^ In this regard, the technique described in this study meets these objectives.

Percutaneous correction of severe forms with provisional K-wire stabilization, a less recent but proven technique, provides the same corrective potential as the more modern MIS techniques, with the advantage of avoiding the need for osteosynthesis material (lower cost and no associated complications) and avoiding the complications associated with open techniques. These are the reasons why we use this technique in our daily practice.

The limitations lie in the retrospective nature of the study, which introduces inherent biases, lacks a control group, and includes only medium-term follow-up. Additionally, the collection of certain parameters that serve as comparative outcomes rather than absolute outcomes also contributes to its limitations. The absence of intra- and interobserver analysis of the measurements and data obtained may also introduce potential bias. Although the fact that all procedures were performed by the same surgeon could be considered a strength of the study, it also introduces limitations regarding the reproducibility and generalizability of the technique and the potential for bias. The treatment of severe cases using this approach should be performed by an operator with thorough mastery of the technique, which may help to optimize clinical outcomes.

The strength of our study lies in the fact that the technique was performed by a single experienced surgeon, using a standardized protocol, and independent data collection and statistical analysis, across a significant number of cases. Moreover, few studies address a strictly percutaneous approach to transverse osteotomy for severe forms, which captures the radiologic corrective efficiency of major but rarely documented parameters such as the correction of the sesamoids and the congruence of the MTPI. Additionally, a detailed analysis of the nature of complications and the association between study parameters enhances the value of this study.

## Conclusion

The correction of severe HV using percutaneous distal metatarsal osteotomy has proven effective, yielding substantial improvements in radiographic alignment, functional outcomes, and patient-reported satisfaction. The absence of major complications, along with the use of temporary fixation without permanent implants, constitutes a notable advantage of this approach. These results support our initial hypothesis that this minimally invasive technique—traditionally reserved for mild and moderate deformities—can be safely and effectively extended to the management of severe cases. This study distinguishes itself in the current literature as one of the largest series dedicated exclusively to severe deformities treated using a strictly standardized percutaneous technique. It offers a detailed assessment of key radiologic parameters—namely, sesamoid position and MTPI joint congruency—and highlights a specific complication, residual soft tissue exostosis, which is rarely discussed in depth in previous studies.

Nonetheless, several limitations must be acknowledged. First, the retrospective design and absence of a control group limit causal inferences. Second, the medium-term follow-up (mean 27.1 months) precludes assessment of long-term durability and recurrence patterns. Third, all procedures were performed by a single experienced surgeon, which may limit generalizability. Finally, some outcome measures rely on nonvalidated patient satisfaction scales, although these are commonly used in hallux valgus literature.

These results support the feasibility and effectiveness of percutaneous distal metatarsal osteotomy for severe hallux valgus, with substantial radiographic improvements and high patient satisfaction. Although these outcomes appear promising compared with historical reports of other techniques, direct comparative studies are needed to establish relative efficacy and safety profiles.

## Supplemental Material

sj-pdf-1-fao-10.1177_24730114251363448 – Supplemental material for Correction of Severe Hallux Valgus Deformity Using a Percutaneous Metatarsal Distal Osteotomy Supplemental material, sj-pdf-1-fao-10.1177_24730114251363448 for Correction of Severe Hallux Valgus Deformity Using a Percutaneous Metatarsal Distal Osteotomy by Jonas Müller, Gilles Dietrich, Igor Gossuin, Marc Prod’homme, Salah Dine Qanadli and Elyazid Mouhsine in Foot & Ankle Orthopaedics
